# *Adolescer Saudável*: screening and follow-up of risk at school

**DOI:** 10.31744/einstein_journal/2021AO5849

**Published:** 2021-09-09

**Authors:** Vânia Leitão Martins, Inês Belo, Alexandra Luz, Pascoal Moleiro

**Affiliations:** 1 Centro Hospitalar Leiria-Pombal Leiria Portugal Centro Hospitalar Leiria-Pombal, Leiria, Portugal.

**Keywords:** Adolescent health, “*Adolescer Saudável*” project, Biopsychosocial risk, Schools, Surveys and questionnaires, Risk

## Abstract

**Objective:**

To characterize adolescents referred to medical consultation based on the screening tool “*Perfil de Saúde do Utente Adolescente*”, and to compare to information gathered from a questionnaire and data assessed during the visit.

**Methods:**

A retrospective and descriptive study, with analysis of the questionnaires filled out by adolescents and their respective medical records, in the period from January 2013 to June 2016.

**Results:**

A total of 54 adolescents were seen, 57% male and mean age of 12±1.7 years. In the questionnaire, 37% stated that they had some kind of health problem; 35% would like to change the relationship with their parents; 18% had some concern about safety at school; and 39% made dietary mistakes. Approximately 31% had consumed alcohol, 13% had tried smoking, and 4% had used other drugs. At the first medical appointment, 38% stated they had chronic disease, 11% reported poor family environment, 39% had school problems and 39% made dietary mistakes. About 13% had tried smoking, 24% had tried to consume alcohol, and 2% had tried other drugs. Thirty seven percent of adolescents were referred to adolescent medicine consultation, and 39% to another hospital consultation.

**Conclusion:**

Many of the biopsychosocial risk items identified through the questionnaire were confirmed during consultation, indicating that it could be a useful screening method for problems linked to the adolescence period.

## INTRODUCTION

Adolescence, as a period of the life cycle, is characterized by physical, psychological, and social change, and can be considered a phase of differentiation, with doubts and conflicts associated with the need for discovery and experimentation.^(1-5)^

Child and adolescent development is influenced by school, family, and friends. School, as an educational entity and place where adolescents spend much of their day, emerges as a privileged environment for prevention of risk behaviors and promotion of healthy lifestyles, as it is one of the most appropriate places for intervention and implementation of projects aimed at health promotion.^(2)^

The *Adolescer Saudável* project, which ran from 2011 to 2016, sought to promote the link between hospital healthcare and the school environment. It took place through the mass application of a validated questionnaire (Appendix 1) containing an adolescent biopsychosocial risk assessment scale called *Perfil de Saúde do Utente Adolescente* (PSUA) [Health Profile of Adolescent Users].^(6)^ After obtaining written informed consent, a team composed of two pediatricians with training in adolescent medicine and two intern physicians with specific training in pediatrics, went to school grounds to perform a medical consultation with adolescents identified as at risk. This project was pioneering in that it sought to conduct a mass screening of adolescents and select those at risk, carrying out their medical assessment in the place where they were daily, and minimizing the impact of adolescents and their families commuting.

Risk to adolescents is characterized as potential danger to the fulfillment of children’s rights in the areas of safety, health, training, education, and development. Risk factors represent physical, psychological, and social variables that may potentiate, in the environment in which they occur, changes that prevent the proper development and socialization of children and youth.^(7)^

Adolescent health, on the other hand, is considered here in accordance with the precept established by the World Health Organization, according to which “health is a state of complete physical, mental, and social well-being and not merely the absence of disease.” During adolescence, it is essential to invest in disease prevention by reducing risk factors, and to promote health with a focus on quality of life, which corresponds to personal satisfaction, development of social skills, and establishment of healthy behaviors.^(8)^

## OBJECTIVE

To characterize the adolescents referred for medical consultation based on the screening tool “*Perfil de Saúde do Utente Adolescente*”, and to compare to information gathered from a questionnaire and data assessed during the visit.

## METHODS

A retrospective and descriptive study was conducted, checking the questionnaires filled out by adolescents and the respective clinical consultation files, carried out in the period between January 2013 and June 2016. Demographic, biopsychosocial risk, diagnosis, and orientation variables were analyzed. Data processing was performed using the (SPSS), version 22.

The target population for this study were all adolescents aged between 10 and 16 years, from 543 students in the second and third cycles (from the fifth to the ninth year of schooling). They were from a public school in the Municipality of Leiria, with informed consent from the legal guardian or from themselves when applicable, favorable to participation. During a first phase, the school teachers/headmasters distributed the questionnaires, which were then delivered to the medical team of the *Centro Hospitalar de Leiria*. After the quantification of the score obtained on the PSUA, a first consultation was scheduled on the school premises, by the same team, for adolescents considered at risk.

A medical evaluation was carried out, and referrals for other consultations in hospital outpatient clinics were made when necessary. It was also possible to consult students referred by teachers/headmasters, or at the request of the adolescents themselves, by filling out the project’s referral form.

The data obtained in screening and those collected at the first visit regarding risk situations identified were analyzed by grouping the topics covered as per a model of adolescent user assessment entitled, Home, Education/Employment, Eating, Activities, Drugs, Sexuality, Suicide/ Depression, and Safety (HEADSSS).^(9)^

The study was approved by the Ethics Committee of the *Centro Hospitalar de Leiria*, EC number. 16/13. Confidentiality, anonymous data, and professional secrecy were preserved, and there were no ethical issues involved.

## RESULTS

Between January 1, 2013 and June 30, 2016, a total of 543 adolescents participated in the study, 53.2% of whom were male, with a mean age of 11.9 years, standard deviation (SD) of 1.6 years, minimum age of 10 years, and maximum age of 16 years.

Fifty-four adolescents (10% of total screened), assessed as at-risk by means of the PSUA, underwent in-school consultations. Of these, 32 (59.3%) were screened directly by the questionnaire, 20 (37%) were given consultations at the request of teachers, and in two (3.7%) cases, the adolescents themselves requested the consultation.

Thirty-one of them (57%) were male adolescents (1.3 M:1 F). The mean age was 12 years, with a SD of 1.7 years, minimum age of 10 years, and maximum of 15 years. There were more adolescents aged between 10 and 12 years (31; 57%), when compared to the group between 13 and 15 years (23; 43%), maintaining the predominance of males in both groups. Regarding the level of schooling, 20 (37%) were fifth grade students, nine (17%) attended sixth grade, 12 (22%) seventh grade, six (11%) eighth grade, and seven (13%) were in ninth grade.

The results of the comparison between the data obtained at screening and those collected in the first consultation are demonstrated in table 1.

**Table 1:** Comparison between the data obtained at screening and at the first medical consultation in the school setting

	Questionnaire containing PSUA	n (%)	Medical consultation in the school setting	n (%)
Medical follow-up	Reported health problems	20 (37)	Had a chronic illness	21 (38)
	Worried about their health	33 (61)	Regular consultations (pediatrician/family doctor)	34 (63)
	Would like to come to a medical appointment	34 (63)		
Dwelling (family)	Would like to change their relationship with their parents	19 (35)	Poor family environment	7 (13)
	Thought their parents did not get along	22 (41)		
Education	“Things” were not going well at school	18 (33)	School problems	21 (39)
Activities /eating	No activity or sport outside of school	21 (39)	No extracurricular activities	19 (35)
	Did not eat “certain meals”	21 (39)	Dietary mistakes	26 (48)
Drugs (usage)	Tobacco	6 (11)	Tobacco	7 (13)
	Alcohol	17 (32)	Alcohol	14 (26)
	Other drugs	2 (4)	Other drugs	1 (2)
Sexuality	Did not know what a STI is	24 (44)	Counseling STI/contraception	25 (46)
	Did not know what contraception is	43 (80)		
Safety	Did not wear a helmet when riding a bike	41 (76)	Did not wear a helmet when riding a bike	41 (76)
	Did not wear a helmet when riding a motorcycle	25 (46)	Did not wear a helmet when riding a motorcycle	20 (37)
	Did not wear seat belts in the car	6 (11)	Did not wear seat belts in the car	3 (6)
	Did not feel safe at school	10 (19)	Did not feel safe at school	
Suicide/ depression/sleep	Had already thought about dying	17 (32)	Thoughts of death (11.1%) / Suicide attempts (3.7%)	8 (15)
	Had difficulty falling asleep	23 (43)	Initial insomnia	16 (30)
	Woke up during the night	19 (35)	Terminal insomnia	5 (9)

PSUA: *Perfil de Saúde do Utente Adolescente*; STI: Sexually Transmitted Infections.

Regarding medical follow-up, in the questionnaire 20 (37%) adolescents reported having some health problem, and 34 (63%) answered that they would like to go to a medical consultation. During the consultation, it was found that 21 (38%) of them had a chronic disease, and 34 (63%) were followed-up with regular consultations. Nineteen (35%) adolescents had no medical follow-up, and the others were followed-up in regular consultations by a pediatrician or family doctor.

When asked about the family environment, 19 (35%) adolescents said they would like to change their relationship with their parents, and 22 (41%) thought there was some kind of conflict between their parents. At the consultation, seven (13%) said there was a poor family environment. Regarding school and the concerns generated by it, in the questionnaire, 18 (33%) said, “things were not going well” at school, and at the consultation, 21 (39%) adolescents revealed school problems. In ten (19%), some kind of concern about safety issues at school was noted (bullying in the school environment, safety of equipment used, ease of help in case of accidents, among others). When diet was questioned, 21 (39%) adolescents revealed that they made dietary mistakes (undiversified and unbalanced diet, excessive intake of fats and sugars, increased intake of soft drinks, low water intake, and excessive dietary restrictions), with the highest number observed in the consultation (26; 48%).

As to consumption, and analyzing the answers given, 17 (32%) adolescents had tried alcohol, 11 (13%) had tried smoking, and two (4%) had tried other drugs, with similar percentages at the medical consultation. When analyzing the results of the questions that addressed safety, namely the use of helmet and seat belt, it was found that 6% and 76% of the adolescents, respectively, did not comply with these measures. The number of young people with altered sleep habits and with thoughts of death varied from 30% to 43%, and 15% to 32%, respectively (Table 1).

**Figure 1:**
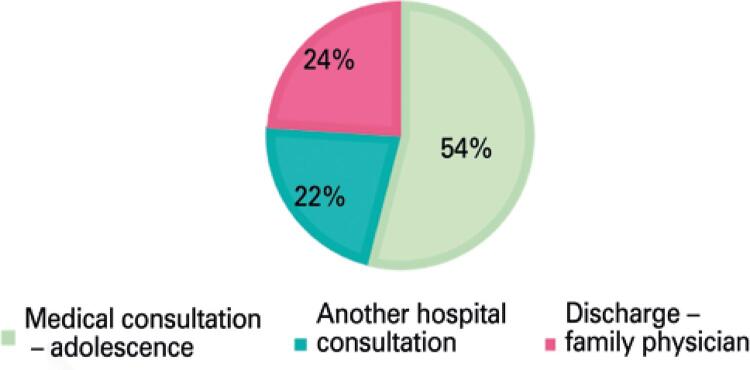
Guidance in hospital consultation

Of all students who attended a first consultation in a school setting, 41 (76%) were referred for a subsequent hospital outpatient consultation. Of these, 29 (54%) were referred for the adolescent medical consultation (69% attended the first consultation), and 12 (22%) to another consultation in a hospital outpatient clinic (Figure 1). At the time this manuscript was prepared, 19 (35%) were followed-up by means of consultations. Among the adolescents who were referred for another consultation in a hospital outpatient clinic, the distribution was as follows: six (50%) were referred for child psychiatry, three (25%) for school pediatrics, one (8%) for general pediatrics, one (8%) for ophthalmology, and one (8%) for oral medicine.

## DISCUSSION

Of the 543 students in the initial target group, 10% were screened for in-school consultation. While this result shows that adolescents are a generally healthy age group,^(2)^ it is worrisome that at least 10% were at biopsychosocial risk. The school environment is ideal for implementing a screening program, as it allows covering all adolescents, not only those referred for consultation.^(10)^

Contrary to what is found in other national studies related to adolescent medicine consultation, in this study, male prevalence was higher,^(2,3,11)^ and literature demonstrates well the fact that boys generally seek health care less.^(1,11-14)^ This study result can be interpreted taking into consideration that we were able to reach the group of boys through the application of the PSUA tool, which allowed us to invite them to a consultation in a school setting.

The number of adolescents referred for consultation in a school environment due to biopsychosocial risk was considerably higher in the younger age groups (between 10 and 12 years), with a predominance of males. This result is in agreement with that already found in another national study,^(3)^ with predominance between 10 and 14 years of age. These results alert us to the fact that risk behaviors start at earlier ages,^(15,16)^which should be taken into account both in individual care and in the definition of public policies aimed at this age group.

Of the 54 adolescents referred to a first consultation in a school environment, in the questionnaire a considerable percentage expressed the wish to go to a medical consultation, and 38% reported having a diagnosed chronic disease (the most frequent, asthma, arrhythmia, visual acuity *deficit*, thyroid pathology, allergic rhinitis and sinusitis), revealing the need for adequate follow-up. In a study where the same questionnaire was applied,^(2)^ the number of young people who wanted to see a physician was slightly lower (45%). This percentage difference may be due, in part, to the fact that in this study only adolescents at biopsychosocial risk were included. But then again, it may be due to a greater need for help on the part of adolescents in the face of earlier exposure to possible risk factors, and a possible increase in the degree of trust in health professionals who went to the school environment.

Another aspect that deserves attention is the percentage of referred adolescents who reported not having any type of medical follow-up (37%), which may reflect an inadequate accompaniment when faced with situations that require it.

When comparing the results obtained using the questionnaire and those evaluated after the first consultation in a school setting, a considerable percentage expressed they would like to change the relationship with their parents. This reveals the difficulty that some families may experience, in the process of both education and autonomy of the adolescent, and in the very interdependence relationship that defines the family context. Other studies show that, during this phase of life, arguments, and disputes between parents and children increase, accompanied by a decline in closeness and companionship.^(16,17)^ In medical consultation, however, the percentage of adolescents who reported “poor family environment” was lower. This number may be related to the fact that, at the time of completing the questionnaire, this situation was in fact verified, however, had been resolved by the time of the consultation. On the other hand, the percentage difference can also be explained by the difficulty in approaching some topics in the context of a medical consultation. As such, the health professional should also direct his efforts towards a visit that captivates the adolescent, *i.e*., be easily accessible (like this one, in a school setting), guaranteeing confidentiality and no prejudice or judgments.^(4)^

As for “school problems,” the answers were quite similar whether by questionnaire or consultation. Although not negligible (33% and 39%, respectively), these values are still lower than those found in a study by Camacho et al.,^(18)^ which was included in the international study Health Behaviour in School-aged Children (HBSC), in which more than half of the respondents (55.5%) reported liking school “more or less,” identifying problems related to this environment. In fact, there seems to be a direct relationship between the school environment and adolescent well-being, which once again alerts to the need for health education in schools. By meeting adolescents’ needs and concerns, school can become a place where adolescents feel well.^(19,20)^

Regarding food, almost half admitted to making some kind of dietary mistakes in the consultation environment, a value higher than that obtained in the questionnaire. Despite the apparent concern in controlling their weight, adolescents are at nutritional risk due to the dietary mistakes they make, and the high percentage of body fat that many present.^(21)^ In a nationwide study,^(11)^ eating disorders were the most frequent mental disorders in consultation. Perhaps the fact that, in the present study, the physician conducted the interview making it more directed and detailed, explains the difference in responses between completion of the questionnaire and the data obtained at the medical appointment.

The answers regarding consumption were also very similar between questionnaire and consultation. There was a higher consumption of alcohol, followed by tobacco and other drugs. These answers agree with the literature.^(2,3,15,22)^ It is also noteworthy that tobacco use by itself may be a predictor of use of other substances; hence, programs directed towards preventing its use may have repercussions on the others.^(14)^ The precocity of consumption (by adolescents between 10 and 15 years of age) is another aspect that alarms in this study.

Although not directly a risk factor, it was worrisome to note that almost half of the sample did not know what a sexually transmitted infection is, and that a high percentage of these adolescents answered they did not know what contraception is. The fact they were predominantly younger adolescents may explain this; on the other hand, it clearly demonstrates the need to reinforce or adapt educational strategies with regard to the sexual education of children and adolescents.

The issues related to safety and sleeping habits were relatively concordant between the questionnaire and the data obtained at the consultation, and once again were a cause for concern. There was a high percentage of adolescents who reported not complying with safety rules (use of helmet, seat belt, etc.), which may be related to the lack of notion of risk inherent to the non-use, and lack of safety education, namely in road safety. There was also a large percentage of adolescents with problems related to sleep habits.

Thoughts of death was mentioned in a large percentage of respondents, and this value was higher in the questionnaire than at the consultation. It is urgent to improve the way we approach this topic, paying more attention to signs that that can make a difference, but are not always easy to interpret (*e.g*. social isolation or loss of interest in activities previously practiced).

In total, 76% of adolescents consulted in a school setting were eventually referred for a hospital outpatient consultation, corroborating the importance and relevance of this type of surveillance in a school setting, as well as the need to act and help these adolescents.

Most of those were referred to the adolescent medicine consultation, which allows for a biopsychosocial approach addressing their needs and concerns, followed by the child psychiatry consultation, and other specialties at a lower percentage. Despite not being the main reason for consultation, the psychological/psychiatric condition was present quite frequently, similar to what has been reported in other studies, and highlighting the need for screening for mental diseases in this age group, particularly at an early age.^(3,8,13)^

Finally, the percentage of these young people who still were followed up at the time of writing this manuscript highlights and appreciates the need for this type of intervention.

## CONCLUSION

The validated tool called *Perfil de Saúde do Utente Adolescente* allowed adolescents to be referred to a first medical consultation held in a school setting, and a significant number of them were followed up at an adolescent medical consultation and/or another hospital outpatient consultation. The intervention carried out and the tool used proved to be a useful and effective means of screening to detect and allow health professionals to guide adolescents at biopsychosocial risk.

## Appendix 1

**Table:** Components of the *Perfil de Saúde do Utente Adolescente* (PSUA)

Item description and factor
**Factor 1 – Motivation for consultation**
Do you feel short of breath or cough a lot with exercise?
Are you worried about your health?
Would you like to come to a medical appointment?
Would you like to know more about sexuality?
**Factor 2 – Physical component**
Have you ever fainted during physical exercise?
Do you feel that you get tired more quickly than your colleagues do?
Do you often wake up during the night?
Do you feel very tired during the day?
Does your stomach often hurt?
Does your head often hurt?
**Factor 3 – Psychological component**
Does it sometimes feel like you are going to faint?
Would you like to change your relationship with your parents?
Do you know someone who felt so sad that they thought about dying?
Have you ever thought about that, too?
Do you have a personal problem that you would rather not write about?
**Factor 4 – Body image**
Do you think you are too fat?
Are there parts of your body that you do not like?
PSUA – Total
PSUA expressed in values between 0 and 10 = least healthy profile; 1 = most healthy profile.
