# Supplementary material for: Adolescer Saudável: screening and follow-up of risk at school
Source: Einstein (Sao Paulo). 2021 Sep 9;19:eAO5849. doi: 10.31744/einstein_journal/2021AO5849 (PMC8428807; doi:10.31744/einstein_journal/2021AO5849)
Supplement: Translation

# Translation: Adolescer Saudável: rastreio e seguimento do risco na escola

**Authors:** Vânia Leitão Martins, Inês Belo, Alexandra Luz, Pascoal Moleiro

**Affiliations:** 1 Portugal Centro Hospitalar Leiria-Pombal, Leiria, Portugal.

**Keywords:** Saúde do adolescente, Projeto adolescer saudável, Risco biopsicossocial, Instituições acadêmicas, Inquéritos e questionários, Risco

## Abstract

**Objetivo:**

Caracterizar adolescentes referenciados à consulta médica a partir do instrumento de triagem Perfil de Saúde do Utente Adolescente e comparar as informações obtidas do questionário e os dados avaliados na consulta.

**Métodos:**

Estudo retrospectivo e descritivo, com análise dos questionários preenchidos por adolescentes e respectivos processos clínicos da consulta realizada no período de janeiro de 2013 a junho de 2016.

**Resultados:**

Foram consultados 54 adolescentes, 57% do sexo masculino, com média de idade 12±1,7 anos. No questionário, 37% responderam ter algum problema de saúde; 35% gostariam de mudar a relação com os pais; 18% tinham algum tipo de preocupação com a segurança na escola; e 39% cometiam erros alimentares. Aproximadamente 31% já tinham consumido álcool, 13% já tinham experimentado fumar, e 4% tinham experimentado outras drogas. Na primeira consulta, 38% dos respondentes responderam ter doença crônica, 11% referiram mau ambiente familiar, 39% apresentaram problemas escolares e 39% revelaram erros alimentares. Cerca de 13% experimentaram fumar, 24% consumiram álcool, e 2% experimentaram outras drogas. Foram referenciados à consulta de medicina do adolescente 37% dos adolescentes, e 39% foram direcionados a outra consulta hospitalar.

**Conclusão:**

Em consulta, confirmaram-se muitos dos itens de risco biopsicossocial identificados por meio do questionário aplicado, o que pode indicar que este é um método útil no rastreio de problemática ligada à adolescência.

## INTRODUÇÃO

A adolescência, enquanto período do ciclo de vida, é caracterizada por mudanças físicas, psicológicas e sociais, podendo ser considerada como uma fase de diferenciação, com dúvidas e conflitos associados à necessidade de descoberta e de experimentação.^(1-5)^

O desenvolvimento do adolescente e da criança é influenciado pela escola, pela família e pelos amigos. A escola, enquanto entidade formativa e local onde os adolescentes passam grande parte do dia, surge como ambiente privilegiado para a prevenção de comportamentos de risco e a promoção de estilos de vida saudáveis, sendo um dos lugares mais adequados para intervenção e realização de projetos que visam à promoção da saúde.^(2)^

O projeto Adolescer Saudável, que decorreu entre 2011 e 2016, procurou promover a articulação entre os cuidados de saúde hospitalares e o meio escolar. Por meio da aplicação em massa de um questionário validado (Apêndice 1), contendo uma escala de avaliação do risco biopsicossocial do adolescente denominada Perfil de Saúde do Utente Adolescente (PSUA),^(6)^ e após consentimento informado, uma equipe constituída por dois pediatras, com formação em medicina do adolescente, e dois médicos internos, de formação específica em pediatria, deslocou-se ao recinto escolar, para efetuar uma consulta médica com adolescentes identificados como de risco. Este projeto foi pioneiro, pois procurou realizar um rastreio em massa de adolescentes e selecionar os que estão em risco, realizando sua avaliação médica no local onde se encontravam diariamente e minimizando o impacto de deslocamentos para os próprios adolescentes e para suas famílias.

Risco para o adolescente configura-se como perigo potencial para a efetivação dos direitos da criança, no domínio da segurança, saúde, formação, educação e desenvolvimento. Os fatores de risco representam variáveis físicas, psicológicas e sociais, que podem potenciar, no meio em que ocorrem, alterações impeditivas do adequado desenvolvimento e da socialização das crianças e dos jovens.^(7)^

Já saúde do adolescente é tomada aqui de acordo com o preceito estabelecido pela Organização Mundial de Saúde, segundo o qual “saúde é um estado de pleno bem-estar físico, mental e social e não apenas a ausência doença”. Durante a adolescência, é essencial investir na prevenção da doença, reduzindo-se os fatores de risco, e promover a saúde, com foco na qualidade de vida, que corresponde à satisfação pessoal, ao desenvolvimento de competências sociais e ao estabelecimento de comportamentos saudáveis.^(8)^

## OBJETIVO

Caracterizar os adolescentes referenciados à consulta médica a partir do instrumento de triagem “Perfil de Saúde do Utente Adolescente” e comparar as informações obtidas do questionário e os dados avaliados na consulta.

## MÉTODOS

Foi feito um estudo retrospectivo e descritivo, com checagem de questionários preenchidos pelos adolescentes e respectivos processos clínicos de consulta, realizado no período entre janeiro de 2013 e junho de 2016. Foram analisadas variáveis demográficas, de risco biopsicossocial, diagnóstico e orientação. O processamento de dados foi realizado com recurso ao programa (SPSS), versão 22.

A população-alvo deste trabalho foram todos os adolescentes entre 10 e 16 anos, num total de 543 alunos do segundo e terceiro ciclos (do quinto ao nono ano de escolaridade), de uma escola pública do Concelho de Leiria, com consentimento informado do responsável legal ou do próprio quando aplicável, favorável à participação. Em uma primeira fase, os professores/diretores de turma distribuíram os questionários, que foram depois entregues à equipe médica do Centro Hospitalar de Leiria. Após quantificação da pontuação obtida no PSUA, foi agendada uma primeira consulta realizada no espaço escolar, pela mesma equipe, para adolescentes considerados de risco.

Foi realizada a avaliação médica, e foi feita referenciação para outras consultas em ambulatório hospitalar, quando necessário. Era igualmente possível consultar alunos que os professores/diretores de turma referenciavam ou a pedido do próprio adolescente, mediante o preenchimento da ficha de referenciação do projeto.

O dados obtidos no rastreio e aqueles conseguidos na primeira consulta relativamente a situações de risco identificadas foram analisados agrupando-se os tópicos abordados segundo modelo de avaliação do utente adolescente *Home, Education/Employment, Eating, Activities, Drugs Sexuality, Suicide/Depression, and Safety* (HEADSSS).^(9)^

O estudo foi aprovado pela Comissão de Ética do Centro Hospitalar de Leiria, CE N.°16/13. Confidencialidade, dados anônimos e segredo profissional foram preservados, e não houve nenhuma questão ética envolvida.

## RESULTADOS

Entre 1^o^ de janeiro de 2013 e 30 de junho de 2016, participaram do estudo 543 adolescentes, dos quais 53,2% eram do sexo masculino, com média de idade de 11,9 anos, desvio-padrão (DP) de 1,6 ano, idade mínima de 10 anos e máxima de 16 anos.

Passaram pela consulta em meio escolar 54 adolescentes (10% do total dos rastreados), avaliados como de risco por meio do PSUA. Destes, 32 (59,3%) foram rastreados diretamente pelo questionário, 20 (37%) passaram pela consulta a pedido de professores e, em dois (3,7%) casos, foi o próprio adolescente que a solicitou.

Constatou-se que 31 (57%) eram adolescentes do sexo masculino (1,3 M:1 F). A média de idade foi de 12 anos, com DP de 1,7 ano, idade mínima de 10 anos e máxima de 15 anos. Verificou-se maior número de adolescentes com idades compreendidas entre os 10 e os 12 anos (31; 57%), quando comparado com o grupo entre os 13 e os 15 anos (23; 43%), mantendo-se o predomínio do sexo masculino em ambos os grupos. Já no que diz respeito ao nível de escolaridade, 20 (37%) eram alunos do quinto ano, nove (17%) frequentavam o sexto ano, 12 (22%) o sétimo ano, seis (11%) o oitavo ano e sete (13%) o nono ano.

O resultado da comparação entre os dados obtidos no rastreio e aqueles conseguidos na primeira consulta estão demonstrados na tabela 1.

**Tabela 1:** Comparação entre os dados obtidos no rastreio e na primeira consulta médica em meio escolar

	Questionário contendo PSUA	n (%)	Consulta médica em meio escolar	n (%)
Acompanhamento médico	Referiam problema de saúde	20 (37)	Tinham doença crónica	21 (38)
	Preocupados com a saúde	33 (61)	Consultas regulares (pediatra/ médico de família)	34 (63)
	Gostavam de vir a uma consulta médica	34 (63)		
Habitação (família)	Gostariam de mudar a relação com os pais	19 (35)	Mau ambiente familiar	7 (13)
	Achavam que os pais não se davam bem	22 (41)		
Educação	“Coisas” não estavam a correr bem na escola	18 (33)	Problemas escolares	21 (39)
Atividades/alimentação	Sem atividade ou desporto fora da escola	21 (39)	Sem atividades extraescolares	19 (35)
	Não faziam “certas refeições”	21 (39)	Erros alimentares	26 (48)
Drogas (consumo)	Tabaco	6 (11)	Tabaco	7 (13)
	Álcool	17 (32)	Álcool	14 (26)
	Outras drogas	2 (4)	Outras drogas	1 (2)
Sexualidade	Não sabiam o que é uma IST	24 (44)	Aconselhamento IST/ contracepção	25 (46)
	Não sabiam o que é contracepção	43 (80)		
Segurança	Não usavam capacete ao andar de bicicleta	41 (76)	Não usavam capacete ao andar de bicicleta	41 (76)
	Não usavam capacete ao andar de moto	25 (46)	Não usavam capacete ao andar de moto	20 (37)
	Não usavam cinto de segurança no carro	6 (11)	Não usavam cinto de segurança no carro	3 (6)
	Não se sentiam seguros na escola	10 (19)	Não se sentiam seguros na escola	
Suicídio/ depressão/sono	Já tinham pensado em morrer	17 (32)	Pensamentos de morte (11,1%) /Tentativa (3,7%)	8 (15)
	Tinham dificuldade em adormecer	23 (43)	Insônia inicial	16 (30)
	Acordavam durante a noite	19 (35)	Insônia terminal	5 (9)

PSUA: Perfil de Saúde do Utente Adolescente; IST: Infeção Sexualmente Transmissível.

No que se refere ao acompanhamento médico, 20 (37%) adolescentes referiram, no questionário, ter algum problema de saúde, e 34 (63%) gostariam de ir a uma consulta médica. Já em consulta, foi constatado que 21 (38%) deles tinham uma doença crônica, e 34 (63%) eram seguidos em consulta regular. Não tinham qualquer seguimento médico 19 (35%) adolescentes, sendo os demais seguidos em consultas regulares por um pediatra ou médico de família.

Quando abordadas questões relativas ao ambiente familiar, 19 (35%) adolescentes disseram que gostariam de mudar a relação com os pais, e 22 (41%) achavam que existia algum tipo de conflito entre os pais. Na consulta, sete (13%) afirmaram haver mau ambiente familiar. Relativamente à escola e as preocupações geradas por ela, no questionário, 18 (33%) responderam que “as coisas não estavam a correr bem” na escola e, na consulta, 21 (39%) adolescentes revelaram problemas escolares. Em dez (19%), foi constatado algum tipo de preocupação com questões de segurança na escola (*bullying* em meio escolar, segurança dos equipamentos utilizados, facilidade de auxílio em caso de acidente etc.). Quando se questionou a alimentação, 21 (39%) adolescentes revelaram cometer erros alimentares (alimentação pouco diversificada e pouco equilibrada, excesso de ingestão de gorduras e açúcares, ingestão aumentada de refrigerantes e fraca ingestão hídrica, além de restrição alimentar excessiva) observando-se número mais elevado na consulta (26; 48%).

No que se refere a consumos e analisando as respostas dadas, 17 (32%) adolescentes tinham experimentado álcool, 11 (13%) já tinham experimentado fumar e dois (4%) tinham experimentado outras drogas, tendo-se verificado percentagens semelhantes em consulta. Quando analisados os resultados das questões que abordavam a segurança, nomeadamente o uso de capacete e cinto de segurança, verificou-se que, respectivamente, 6% e 76% dos adolescentes não cumpriam essas medidas. A percentagem de jovens com alterações dos hábitos de sono e com pensamentos de morte foi, respectivamente, de 30% a 43% e de 15% a 32% (Tabela 1).

De todos os alunos que foram a uma primeira consulta em meio escolar, 41 (76%) foram referenciados a uma consulta subsequente em ambulatório hospitalar. Destes, 29 (54%) foram referenciados à consulta de medicina do adolescente (69% compareceram à primeira consulta) e 12 (22%) a outra consulta em ambulatório hospitalar (Figura 1). No momento da redação deste trabalho, mantinham seguimento em alguma consulta 19 (35%) deles. Dentre os adolescentes que foram referenciados para outra consulta em ambulatório hospitalar, a distribuição foi a seguinte: seis (50%) foram referenciados para a pedopsiquiatria, três (25%) para a pediatria escolar, um (8%) para a pediatria geral, um (8%) para a oftalmologia e um para a (8%) estomatologia.

**Figura 1:** Orientação na consulta em meio hospitalar

## DISCUSSÃO

Dos 543 alunos que pertenciam ao grupo-alvo inicial, 10% foram rastreados para a consulta em meio escolar. Se por um lado, este resultado nos demonstra que os adolescentes são um grupo etário geralmente saudável,^(2)^ por outro é preocupante verificar que pelo menos 10% estavam em risco biopsicossocial. O ambiente escolar é ideal para implementar um programa de rastreio, pois permite abranger todos os adolescentes, e não apenas os referenciados à consulta.^(10)^

Neste estudo, foi maior a prevalência do sexo masculino, ao contrário do que se verifica noutros estudos nacionais relacionados com a consulta de medicina da adolescência.^(2,3,11)^ Está igualmente bem demonstrado na literatura que os rapazes geralmente procuram menos os cuidados de saúde.^(1,11-14)^ Tal resultado neste estudo pode ser interpretado considerando-se o fato de se ter conseguido chegar ao grupo dos rapazes por meio da aplicação do instrumento PSUA, o que permitiu convocá-los para uma consulta em meio escolar.

O número de adolescentes referenciado para consulta em meio escolar por risco biopsicossocial foi consideravelmente maior nas faixas etárias mais baixas (entre os 10 e 12 anos), mantendo-se o predomínio do sexo masculino. Este resultado é concordante com o já encontrado noutro estudo nacional,^(3)^ com predomínio entre os 10 e os 14 anos. Com esses resultados, alerta-se para o fato de os comportamentos de risco se iniciarem em idades mais precoces,^(15,16)^ o que se deve ter em conta tanto no atendimento individual como na definição de políticas públicas destinadas a esse grupo etário.

Dos 54 adolescentes referenciados a uma primeira consulta em ambiente escolar, percentagem considerável manifestou no questionário a vontade de ir a uma consulta médica, e 38% referiram ter uma doença crônica diagnosticada (sendo as mais frequentes asma, arritmia, *deficit* de acuidade visual, patologia tiroidea, rinite alérgica e sinusite), revelando a necessidade de um seguimento adequado. Num estudo em que foi aplicado o mesmo questionário,^(2)^ o número de jovens que desejava ir a uma consulta médica foi ligeiramente inferior (45%). Essa diferença percentual pode ser devida, por um lado, ao fato de, neste estudo, terem sido incluídos apenas adolescentes em risco biopsicossocial ou, por outro, por uma maior necessidade por parte dos adolescentes em obter ajuda perante uma mais precoce exposição a eventuais fatores de risco e ao possível aumento do grau de confiança nos profissionais de saúde que se deslocaram ao ambiente escolar.

É ainda de se salientar outro aspecto que deve merecer atenção: a percentagem de adolescentes referenciados que referiu não ter qualquer tipo de seguimento médico (37%), o que pode traduzir um acompanhamento desadequado perante situações que dele necessitam.

Quando se comparam os resultados obtidos utilizando-se o questionário e os avaliados após a primeira consulta em contexto escolar, verificou-se ser considerável a percentagem que gostaria de mudar a relação com seus pais, revelando-se a dificuldade que algumas famílias podem apresentar, quer no processo de educação e autonomia do adolescente, quer na própria relação de interdependência que define o contexto familiar. Outros estudos mostram que, durante essa fase da vida, aumentam as discussões e as disputas entre pais e filhos, as quais são acompanhadas pela diminuição da proximidade e do convívio.^(16,17)^ Em consulta médica, no entanto, foi menor a percentagem de adolescentes que referiu existir “mau ambiente familiar”. Esse número pode estar relacionado com o fato de, no momento do preenchimento do questionário, verificava-se, de fato, essa situação, que foi, entretanto, resolvida até a data da consulta. Por outro lado, a diferença de percentagem também pode ser explicada pela dificuldade em abordar alguns temas em contexto de consulta. Como tal, o profissional de saúde deve também direcionar seus esforços para a realização de uma consulta que cative o adolescente, isto é, de fácil acesso (como esta, em meio escolar), com a garantia da confidencialidade e sem caráter preconceituoso e nem julgamentos.^(4)^

Já no que se referiu a “problemas escolares”, as respostas foram bastante semelhantes por meio do questionário ou em consulta. Apesar de não desprezíveis (33% e 39%), estes valores são ainda inferiores aos encontrados num trabalho de Camacho et al.,^(18)^ inserido no estudo internacional do *Health Behaviour in School-aged Children* (HBSC), em que mais de metade dos inquiridos (55,5%) referiu gostar “mais ou menos” da escola, identificando problemas relativos a esse ambiente. De fato, parece haver uma relação direta entre o ambiente escolar e o bem-estar do adolescente, o que alerta, mais uma vez, sobre a necessidade de educação para a saúde nas escolas. Satisfazendo as necessidades e preocupações dos adolescentes, a escola pode se tornar um local onde os adolescentes se sintam bem.^(19,20)^

Relativamente à alimentação, quase metade admitiu cometer algum tipo de erro alimentar em ambiente de consulta, valor mais elevado do que o obtido no questionário. Apesar da aparente preocupação em controlar seu peso, os adolescentes estão sob risco nutricional, pelos erros alimentares que cometem, e pela elevada percentagem de gordura corporal que muitos apresentam.^(21)^ Num estudo nacional,^(11)^ os transtornos alimentares foram as perturbações mentais mais frequentes em consulta. Talvez o fato de, no presente estudo, ser o médico a realizar a entrevista, tornando-a mais dirigida e pormenorizada, justifique a diferença de respostas entre o preenchimento do questionário e os dados obtidos na consulta médica.

Também as respostas relativas aos consumos se verificaram muito semelhantes entre questionário e consulta. Verificou-se maior consumo de álcool, seguido do tabaco e de outras drogas. Essas respostas são concordantes com a literatura.^(2,3,15,22)^ Salienta-se, ainda, que o consumo de tabaco, por si só, pode ser um preditor do consumo de outras substâncias, de modo que programas dirigidos à prevenção do seu uso podem repercutir nos demais.^(14)^ A precocidade nos consumos é também um aspecto que neste estudo (com adolescentes entre os 10 e os 15 anos) alarma.

Apesar de não ser diretamente um fator de risco, foi preocupante constatar que quase metade da amostra não sabia o que é uma infecção sexualmente transmissível, e que elevada percentagem desses adolescentes respondeu desconhecer o que é a contracepção. O fato de serem adolescentes predominantemente mais novos pode explicar esse dado; por outro lado, isso demonstra claramente a necessidade de reforçar ou adaptar estratégias educacionais no que diz respeito à educação sexual de crianças e adolescentes.

As questões relacionadas com a segurança e com os hábitos de sono foram relativamente concordantes entre o questionário e os dados obtidos em consulta, sendo mais uma vez motivo de preocupação. Existiu uma grande percentagem de adolescentes que referiu não cumprir as normas de segurança (uso de capacete, cinto de segurança etc.), o que pode estar relacionado com a falta de noção de risco inerente ao não uso e de educação para a segurança, nomeadamente rodoviária. Foi também elevada a percentagem de adolescentes com problemas relacionados com a higiene do sono.

A presença de pensamentos de morte foi referida numa grande percentagem dos inquiridos, sendo este valor mais elevado no questionário que na consulta. É urgente que se procure melhorar a forma de abordagem desse tema, dando mais atenção a sinais que nem sempre são fáceis de interpretar (por exemplo, o isolamento social ou a perda de interesse por atividades que praticavam), mas que podem fazer a diferença.

No total, acabaram por ser referenciados a uma consulta em ambulatório hospitalar 76% dos adolescentes consultados em ambiente escolar, corroborando a importância e a pertinência desse tipo de vigilância em meio escolar, bem como a necessidade de atuar e de ajudar esses adolescentes.

A maioria foi referenciada à consulta de medicina da adolescência, que permite uma abordagem biopsicossocial, dirigida às suas necessidades e inquietações, seguindo-se a consulta de pedopsiquiatria e as outras especialidades em menor percentagem. Apesar de não ser o principal motivo de consulta, a patologia do foro psicológico/psiquiátrico estava presente de forma bastante frequente, indo de encontro ao referido noutros estudos e salientando a necessidade do rastreio da doença mental nesta faixa etária nomeadamente em idades precoces.^(3,8,13)^

Por fim, a percentagem destes jovens que ainda mantinha seguimento no momento da redação desta pesquisa realça e valoriza a necessidade desse tipo de intervenção.

## CONCLUSÃO

O instrumento validado denominado Perfil de Saúde do Utente Adolescente permitiu que adolescentes fossem referenciados a uma primeira consulta médica realizada em meio escolar, e número significativo deles manteve seguimento em consulta de medicina do adolescente e/ou outra consulta ambulatorial hospitalar. A intervenção realizada e o instrumento utilizado demonstraram ser um meio de rastreio útil e eficaz para detectar e permitir ao profissional de saúde orientar adolescentes em risco biopsicossocial.

## Apêndice 1

**Table:** Perfil de Saúde do Utente Adolescente (PSUA) componentes

Descrição do item e fator
**Fator 1 – Motivação para consulta**
Sentes falta de ar ou tosses muito com o exercício físico?
Estás preocupado/a com a tua saúde?
Gostavas de vir a uma consulta médica?
Gostavas de saber mais coisas sobre sexualidade?
**Fator 2 – Componente físico**
Alguma vez desmaiaste durante o exercício físico?
Sentes que te cansas mais rapidamente do que os teus colegas?
Acordas muitas vezes durante a noite?
Sentes-te bastante cansado durante o dia?
Dói-te muitas vezes a barriga?
Dói-te muitas vezes a cabeça?
**Fator 3 – Componente psicológico**
Às vezes parece que vais desmaiar?
Gostavas de mudar a tua relação com os teus pais?
Conheces alguém que se sentia tão triste que pensava em morrer?
Já pensaste nisso também?
Tens algum problema pessoal que prefiras não escrever?
**Fator 4 – Imagem corporal**
Achas-te muito gordo/a?
Há partes do teu corpo das quais não gostas?
PSUA – Total
PSUA expresso em valores entre 0 e 10 = perfil menos saudável; 1 = perfil mais saudável.
